# Defining the Extracellular Matrix of Rhabdomyosarcoma

**DOI:** 10.3389/fonc.2021.601957

**Published:** 2021-02-23

**Authors:** Xiaolei Lian, J. Steffan Bond, Narendra Bharathy, Sergei P. Boudko, Elena Pokidysheva, Jack F. Shern, Melvin Lathara, Takako Sasaki, Teagan Settelmeyer, Megan M. Cleary, Ayeza Bajwa, Ganapati Srinivasa, Christopher P. Hartley, Hans Peter Bächinger, Atiya Mansoor, Sakir H. Gultekin, Noah E. Berlow, Charles Keller

**Affiliations:** ^1^ Pediatric Cancer Biology, Children’s Cancer Therapy Development Institute, Beaverton, OR, United States; ^2^ Department of Pathology, Oregon Health & Science University, Portland, OR, United States; ^3^ Division of Nephrology and Hypertension, Vanderbilt University Medical Center, Nashville, TN, United States; ^4^ Pediatric Oncology Branch, Center for Cancer Research, National Institutes of Health, Bethesda, MD, United States; ^5^ Bioinformatics, Omics Data Automation, Beaverton, OR, United States; ^6^ Department of Matrix Medicine, Oita University, Oita, Japan; ^7^ Department of Anatomic & Clinical Pathology, Mayo Clinic, Rochester, MN, United States; ^8^ Department of Biochemistry and Molecular Biology, Shriners Hospital for Children, Portland, OR, United States

**Keywords:** matrix, COL18A1, PLOD1/2, rhabdomyosarcoma, survival

## Abstract

Rhabdomyosarcoma (RMS) is the most common soft-tissue sarcoma of childhood with a propensity to metastasize. Current treatment for patients with RMS includes conventional systemic chemotherapy, radiation therapy, and surgical resection; nevertheless, little to no improvement in long term survival has been achieved in decades—underlining the need for target discovery and new therapeutic approaches to targeting tumor cells or the tumor microenvironment. To evaluate cross-species sarcoma extracellular matrix production, we have used murine models which feature knowledge of the myogenic cell-of-origin. With focus on the RMS/undifferentiated pleomorphic sarcoma (UPS) continuum, we have constructed tissue microarrays of 48 murine and four human sarcomas to analyze expression of seven different collagens, fibrillins, and collagen-modifying proteins, with cross-correlation to RNA deep sequencing. We have uncovered that RMS produces increased expression of type XVIII collagen alpha 1 (COL18A1), which is clinically associated with decreased long-term survival. We have also identified significantly increased RNA expression of COL4A1, FBN2, PLOD1, and PLOD2 in human RMS relative to normal skeletal muscle. These results complement recent studies investigating whether soft tissue sarcomas utilize collagens, fibrillins, and collagen-modifying enzymes to alter the structural integrity of surrounding host extracellular matrix/collagen quaternary structure resulting in improved ability to improve the ability to invade regionally and metastasize, for which therapeutic targeting is possible.

## Introduction

Rhabdomyosarcoma (RMS) are highly malignant tumors known to phenocopy some of the early events in skeletal muscle embryogenesis but are also known to arise from tissues not known to contain striated muscle or muscle stem cells (1). Embryonal rhabdomyosarcoma (eRMS) and alveolar rhabdomyosarcoma (aRMS) are the major histological subtypes. eRMS is the most common subtype accounting for half of all RMS cases and occurs most often in the head, neck, and genitourinary tract (2). aRMS usually occurs in adolescents and young adults and is commonly found in the trunk and extremities.

**Figure 1:**
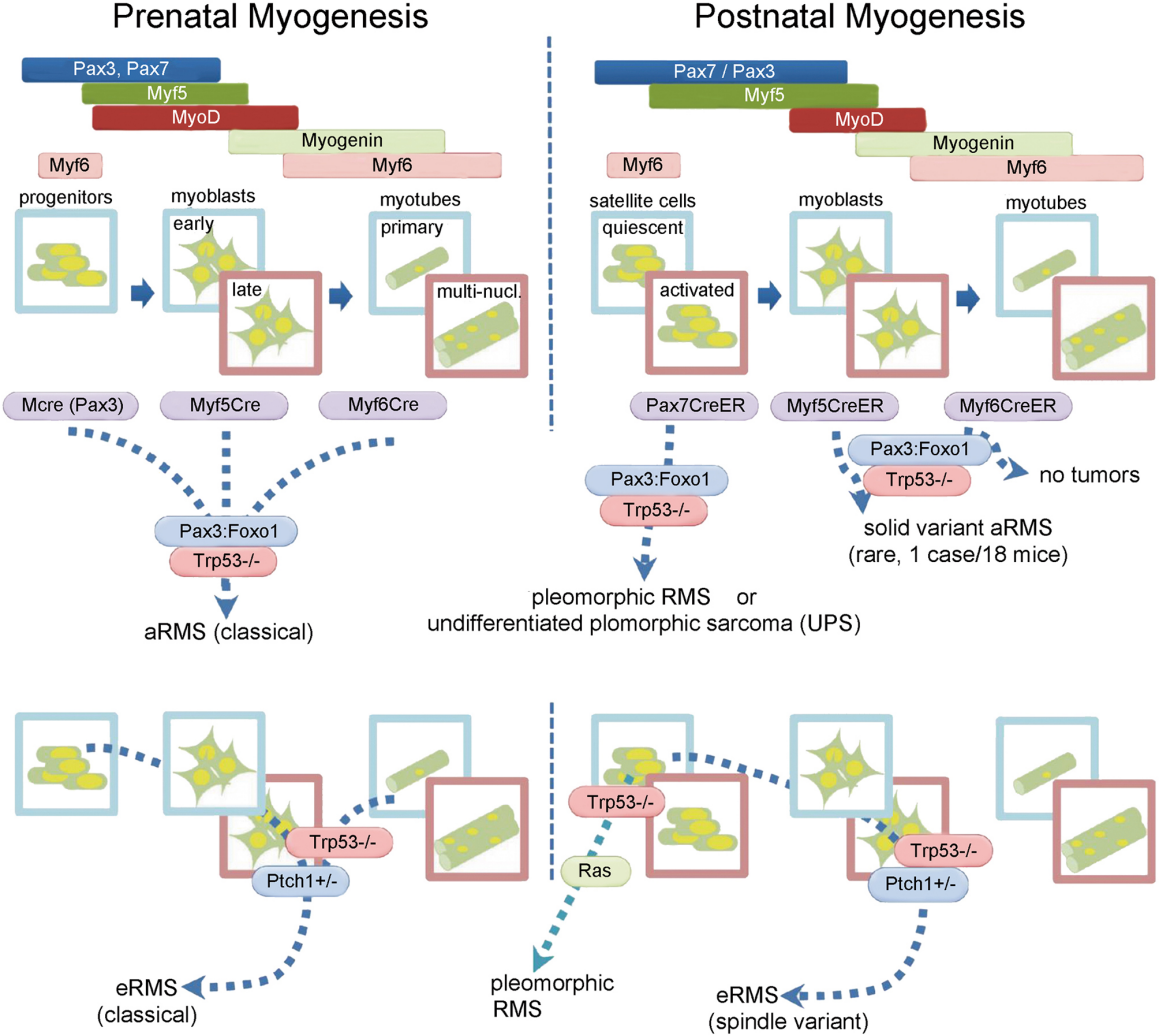
Conditional (Cre/LoxP) Mouse Models of Rhabdomyosarcoma (RMS) and Non-Rhabdomyosarcoma Soft Tissue Sarcomas, including like undifferentiated pleomorphic sarcoma (UPS). Phenotype of the sarcoma depends not only on mutational profile, but also on cell (lineage) of origin and timing of the initiation before or after birth (5–8). Surprisingly, eRMS, and UPS exist in a continuum (7). Also, to our surprise postnatal muscle progenitors almost never gave rise to aRMS.

To address the pressing and unmet clinical need for new treatments of soft tissue sarcomas, we have generated multiple genetically-engineered mouse (GEM) models of aRMS, eRMS and the undifferentiated pleomorphic sarcoma (UPS) subtype of non-rhabdomyosarcoma soft-tissue sarcoma (NRSTS) (3–7) (**Figure 1**). Lymphatic and hematogenous (pulmonary) metastasis is a predominant feature (4) and a primary cause of mortality in these models (9). We characterized these soft tissue sarcoma models and demonstrate them to be representative of the human diseases by histopathology, gene expression and other features (3, 4, 8, 10). Furthermore, these conditional models have the special features of knowing the cell-of-origin as well as the mutational profile making them a valuable tool to study RMS (**Figure 1**).

Very little is known about the composition of the RMS tumor microenvironment (TME). In this study, we have uncovered that type XVIII collagen alpha 1 (encoded by *COL18A1*) is expressed highly in aRMS and eRMS. Interestingly, the variant of rhabdomyosarcoma cell cultures that produce COL18A1 is the ‘alveolar’ subtype, thus called because rich collagen stroma encasing the tumor cells is reminiscent of lung histology.

In addition to analyzing the expression of collagens in RMS, we also examined the expression of the post-translational collagen-modifying enzymes such as prolyl 3-hydroxylase (P3H2, *LEPREL1*), lysyl hydroxylase 1 (LH1, *PLOD1*) and lysyl hydroxylase 2 (LH2, *PLOD2*) which facilitate collagen maturation (reviewed in (11)). Whereas prolyl 4-hydroxylation of the Yaa position of Gly-Xaa-Yaa by P4H enzyme is ubiquitous and provides stability to the collagen triple helix, prolyl 3-hydroxylation by P3H2 in the Xaa position of a Gly-Xaa-4Hyp sequence can be far less frequent—yet biologically essential for processes such as the maternal-fetal interface with respect to collagen IV (12). Like type IV collagen, type XVIII collagen (COL18) contains a relatively large number of Gly-Pro-Hyp sequences (13). Lysyl hydroxylases target the Yaa position lysine residues in Gly-Xaa-Lys tripeptides (11). This post-translational lysyl hydroxylation allows subsequent glycosylation of hydroxylysine. Hydroxylysines and O-linked glycosylation of hydroxylysines within procollagen are essential to the formation of intra- and inter-molecular crosslinks (11). The critical underlying biology of the extracellular matrix not only has implications for histological appearance, but may also create therapeutic opportunities (14), which motivated this study.

## Materials and Methods

### Murine RMS Cell Cultures

All murine cell cultures were isolated from tumors generated in house by our genetically engineered mouse model (GEMM). Isolation of primary tumor cells and culturing method have been previously described (15, 16). Briefly, tumors are dissected from mice under sterile conditions. Tumors are digested over night at 4°C in a 1% collagenase IV solution (17104019, Sigma Aldrich, St Louis, MO, USA) diluted in DMEM. After 18 h, cells are briefly vortexed then passed through a 70 mM filter. Cells were centrifuged at 200 g for 5 min, and the cell pellet was resuspended in 13 mL growth media then transferred to a T75 tissue culture flask. Cells were expanded at 37°C in 5% CO_2_.

Tumor cultures U21459 and U23674 have been previously described (5). U48484 has been previously described (17). PCB-00380 has been previously described (18). PCB-00232 has been previously described (19). The genotype of GEMM tumors used in HIC studies are as follows: U24988-MCreTg Cre/WT, P3Fm/P3Fm, F2-10/F2-10; U20745-MCreTg (Cre/WT), F2-10/F2-10; U37125-Myf6 ICNm/WT, F2-10/F2-10; U34278-Pax7 CreERp/WT, F2-10/F2-10; U24085-Myf5 ICNm/WT, F2-10/F2-10; U24055-Myf5 ICNm/WT, F2-10/Del2-10; U33915: Pax7 CreERp/WT, F2-10/F2-10; U57810- Myf6 ICNm/ICNm, F2-1-/F2-10; U34279- Pax7 CreERp/WT, F2-10/F2-10.

### Immunoblotting

For the analysis of secreted proteins, the medium was collected and proteins were precipitated with 0.2 μg/mL ammonium sulfate, centrifuged, dissolved in Tris Buffered Saline (TBS), and run on the SDS-PAGE. Western blotting with antibodies against COL18A1 (anti-NC1, 1087+ (20); anti-NC11 1112+ obtained from Dr. Takako Sasaki (unpublished)) was performed after transfer to a nitrocellulose membrane.

Cells were also fixed in methanol and stained with rabbit polyclonal antibody against murine type IV collagen from PF-HR9 cells (21), rabbit polyclonal against murine NC2 domain of collagen XVI (trimeric form, native conformation) purified using antigen-coupled column from serum (Hans Peter Bächinger’s Laboratory, unpublished), antibodies against murine COL18A1 (anti-NC1, 1087+; anti-NC11, 1112+ from Dr. Takako Sasaki) and antibody against murine fibrillin-1 (9543 from Dr. Lynn Sakai, Oregon Health & Science University).

The Anti-NC11 antibody has not yet been published, though an associated manuscript is currently in review, and a general method is provided. Polyclonal rabbit anti-NC11 antibody was raised as follows: the RNA isolated from the mouse endothelioma cell line eEnd.2 was used to amplify reverse transcriptase PCR to obtain complementary DNA (cDNA) encoding the TSP1-like domain (NC11) of mouse collagen XVIII. The amplified cDNA was inserted into the episomal expression vector pCEP-Pu, which contained in addition the signal peptide of BM-40, to transfect human EBNA-293 cells (22). The recombinant TSP1 domain was purified from conditioned medium using dimethylaminoethanol (DEAE) cellulose followed by Superose 6 molecular sieve chromatography and then used for immunizing of a rabbit. The specificity of all the anti-NC11 antibody was demonstrated by a complete lack of staining with tissues from Col18a1^−/−^ mice.

### Tissue Microarrays

Four samples of formalin-fixed paraffin embedded (FFPE) human RMS were available to include in a custom mouse model tissue microarray (TMA) (1× aRMS, 3× eRMS). Forty-eight murine model sarcomas were also included, representing developmental stages and genotypes including early myoblast (origin), postnatal stem cell (origin), maturing myofiber (origin), *Pax3:Foxo1*-expressing, *Trp53* wild type or mutated and *Rb1* wildtype or mutated (5, 7, 8). Four samples of murine normal skeletal muscle were included in a custom mouse model tissue microarray (TMA) as the negative control.

The TMA was stained with a standard H&E stain for histologic verification. Co-author AM classified each tumor as non-rhabdomyosarcoma and rhabdomyosarcoma. The latter was further divided into aRMS, eRMS, pleomorphic RMS and RMS not otherwise specified (RMS NOS). We have included a detailed spreadsheet file as Supplementary Table that includes demographic features of each mouse tumor sample present on the TMA (**Supplementary Table 1**). This TMA is publicly available by request.

### Immunohistochemical Staining

All seven IHC stains were performed on the TMA by an immunoperoxidase technique using the following commercial antibodies: anti-COL18A1 (Cat #LS-B8215, rabbit polyclonal, 1:50, LifeSpan Biosciences, Seattle, WA, USA), anti-PLOD1 (Cat #LS-C163796, rabbit polyclonal, 1:100, LifeSpan Biosciences), anti-PLOD2 (Cat #LS-B9694, rabbit polyclonal, 1:100, LifeSpan Biosciences), anti-FBN1 (Cat #LS-B5512, mouse monoclonal, clone 26, 1:400, LifeSpan Biosciences), anti-FBN2 (Cat #LS-B6338, rabbit polyclonal, 1:400, LifeSpan Biosciences), anti-COL4A1 (Cat #LS-C175972, rabbit polyclonal, 1:50, LifeSpan Biosciences) and anti-COL4A2 (Cat # LS-C176967, rabbit polyclonal, 1:50, LifeSpan Biosciences). Histology was conducted using standard protocol. Briefly, Paraffin-embedded 5-μm thick tissue sections were dewaxed and dehydrated in xylene and graded alcohol concentrations and rinsed with 10 mM Tris-HCl at pH 7.4. Slides were then treated with 3% hydrogen peroxide. Slides were subsequently placed in 10 mM citrate buffer (pH 6.0) at 100°C for 20 min. After slides were washed with PBS and incubated at room temperature in 5% normal goat serum (Invitrogen) and 0.01% Triton-X in PBS for 1 h to inhibit non-specific binding of antibodies and incubated with the titrated primary antibody overnight at 4°C, slides were washed with PBS. Signal detection was performed by incubating in biotinylated secondary antibody and subsequently with streptavidin-HRP, and developing in diaminobenzidine solution. Slides were counterstained with hematoxylin for 5 min, rinsed, dehydrated, and mounted with xylene-based mounting medium. Control slides with areas of positive and negative staining are given in **Supplementary Figure 1**.

Each IHC stain was scored by co-author SHG for intensity and percentage of positive cells in each individual tumor sample (some tumors were represented more than once in the TMA). The intensity of each stain was scored accordingly: 0-negative; 1-indeterminate; 2-weak positive; 3-strong positive. The intensity and percentages were averaged for all cases containing more than one TMA fragment. Scoring of intensity was further simplified in a binary scheme where average intensity of greater than 1.5 was considered positive, and 1.5 or less was considered negative.

### Immunocytochemistry Staining

Cells were allowed to recover post sort in culture for 2 to 3 days then replated in eight chamber slides at low confluency and allowed to incubate for 2 to 3 days. Cytology was conducted using standard protocol. Briefly, Cells were then fixed in 4% paraformaldehyde in PBS at room temperature for 20 min. After protein and streptavidin/biotin blocking steps, cells were washed again, and primary antibody diluted in 5% NGS in PBS was added overnight at 4°C. Alexafluor 546 conjugation-based antibody (Invitrogen) at 1:200 was added, and cells were incubated for 1 h at room temperature. Slides were mounted using the VECTASHIELD Mounting Medium with 40, 6-diamidino-2-phenylindole (Vector Laboratories) and visualized with a Zeiss LSM 700 confocal microscope.

### RNA Sequencing Analysis

Tissue for RNA sequencing experiments were obtained from flash frozen tissue samples obtained from biopsy or autopsy tissue (for human subjects) or from flash frozen tumor tissues preserved following necropsy (for murine models). All human tumor tissues were obtained through the pediatric tumor tissue banking program denoted CureFAST following consent by patient and/or patient’s guardians. The CureFAST program is overseen by the Independent Review Board (IRB) that oversees all human-related research at the Children’s Cancer Therapy Development Institute.

Raw transcriptome sequencing data as short read fastq files were aligned to the GRCh38 human reference genome for human data and GRCm38 murine reference genome for mouse data using STAR aligner (23), and aligned transcripts were subsequently quantified for overall expression using RSEM (24). Normal tissue gene expression data matched to individual samples was generally unavailable, thus region-specific skeletal muscle tissue gene expression data was accessed from the Genotype-Tissue Expression (GTEx) project to serve as a population normal and enable comparative expression analysis (25). Sequencing data availability information is provided in the Data Availability statement.

### Statistical Analysis

Binary immunohistochemistry expression values were compared between histologic groups using the Fisher exact test. Statistical analyses were performed using R statistical software (version 3.3.1; R Foundation, Vienna, Austria) with the R commander graphical user interface.

RNA expression comparisons between histologic groups were analyzed using one-way analysis of variance (ANOVA) for cohort comparisons, and individual comparisons between relevant groups (human tumor *vs*. human cell line, human tumor *vs*. human normal, human cell line *vs*. human normal, murine tumor *vs*. murine normal) were made using the two-stage linear step-up procedure FDR adjustment of Benjamini, Krieger and Yekutieli for multiple comparisons. Statistical significance was set at *p < 0.05, **p < 0.01, ***p < 0.001, ****p < 0.0001. Error bars indicate mean ± SD or SEM. Statistical analysis was performed with GraphPad Prism 7.0.

## Results

### COL18A1 Expression Is Elevated in RMS

**Figure 2:**
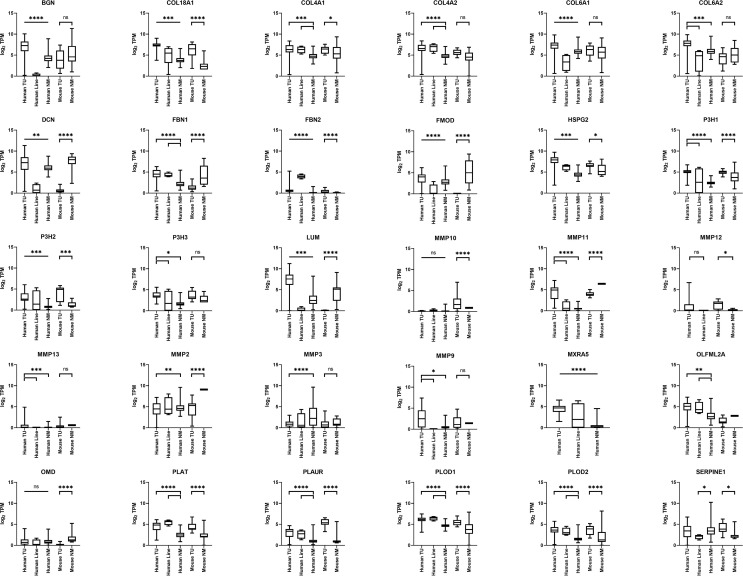
Extracellular Matrix Gene Expression (ECM) gene expression in mouse and human aRMS. Boxplots are given for biopsies versus cell lines versus normal muscle (n = 35 human aRMS biopsies, n = 4 human aRMS cell lines, n = 564 human normal skeletal muscle, n = 13 mouse necropsy aRMS tumors, n = 12 mouse normal skeletal muscle).

**Figure 3:**
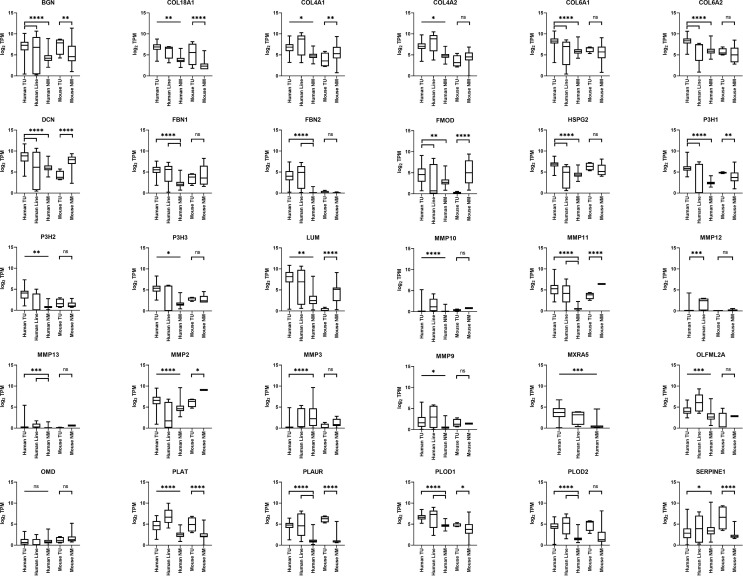
ECM gene expression in mouse and human eRMS. Boxplots are given for biopsies *versus* cell lines versus normal muscle (n = 57 human eRMS biopsies, n = 5 human eRMS cell lines, n = 564 human normal skeletal muscle, n = 4 mouse necropsy eRMS tumors, n = 12 mouse normal skeletal muscle).

**Figure 4:**
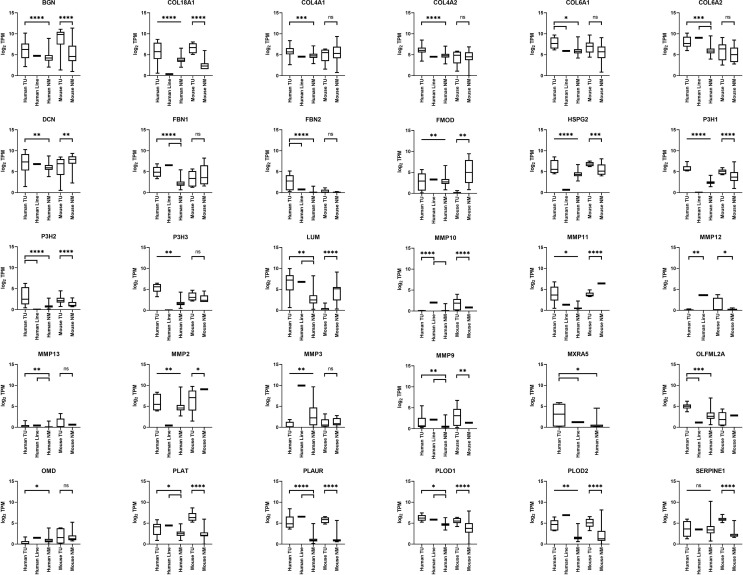
ECM gene expression in mouse NRSTS. Boxplots are given for biopsies *versus* normal muscle (n = 6 mouse necropsy NRSTS tumors, n = 12 mouse normal skeletal muscle).

*COL18A1* showed significantly increased expression at the mRNA level in human and murine aRMS and eRMS tumors relative to normal muscle (**Figures 2** and **3**). *COL4A1*, *FBN1*, and *FBN2* also showed significant overexpression *versus* normal muscle in both human aRMS and eRMS, and *COL4A1* was significantly expressed in murine aRMS (**Figures 2** and **3**). Fibrillin-1 (FBN1) and fibrillin-2 (FBN2) are two subtypes of the fibrillin glycoprotein incorporated into elastic tissue in the extracellular matrix, and FBN2 is a known immunohistochemical biomarker of eRMS (26), which was reflected at the RNA level for eRMS more so than aRMS. Increased expressions of *COL4A2*, *PLOD1*, and *PLOD2* were seen for human RMS tumors (**Figures 2** and **3**). For NRSTS, Col18a1 and Plod2 (but not *Fbn1* and *Fbn2*) were elevated in mouse tumors (**Figure 4**). Statistical tests comparing RNA expression values are presented in **Supplementary Table 2**.

**Figure 5:**
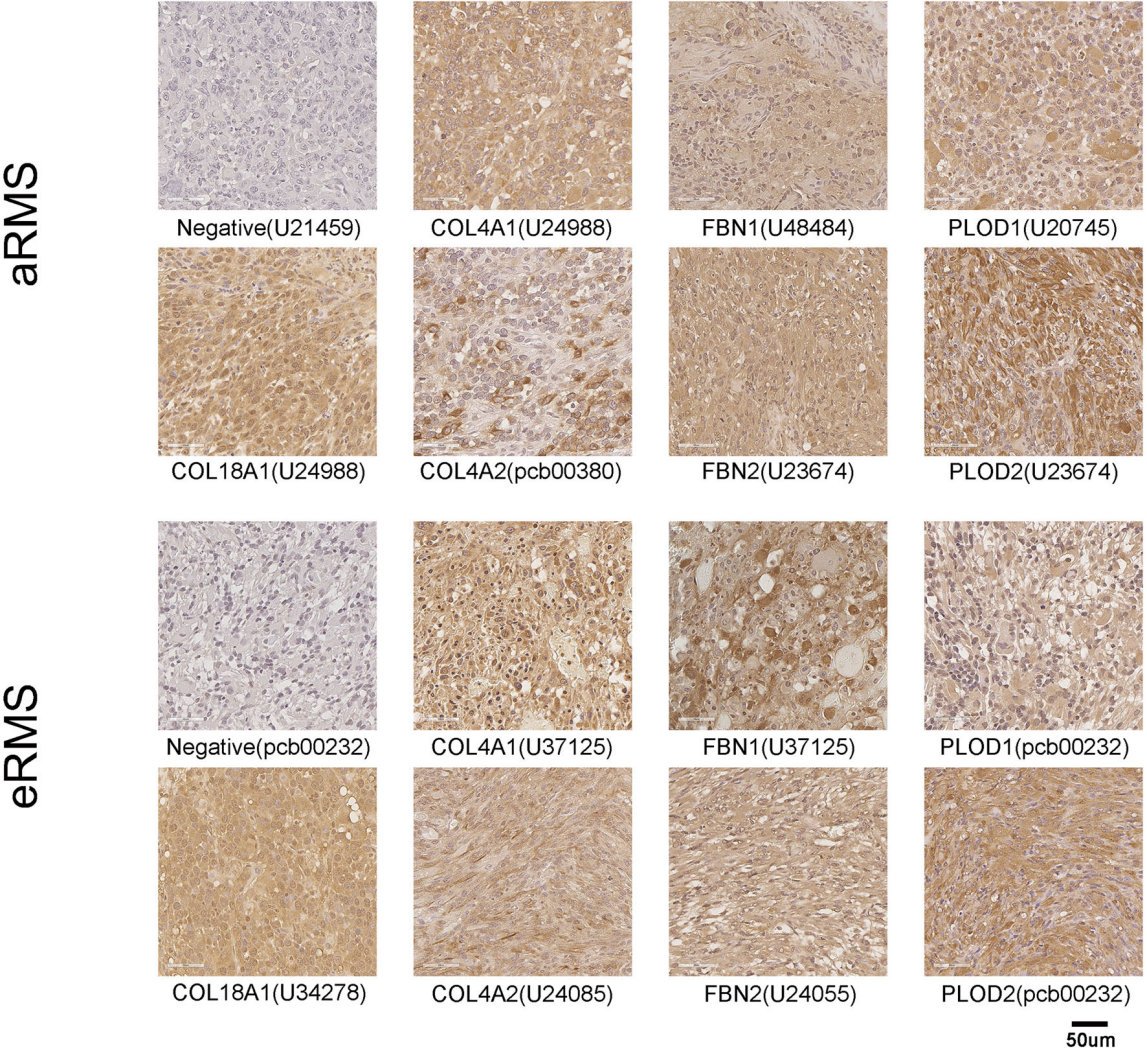
Protein expression of collagens, fibrillins and collagen modifying enzymes in murine aRMS and eRMS. Representative images showing tissue microarray negative control immunohistochemical staining and positive tumoral immunohistochemical staining of COL18A1, COL4A1, COL4A2, FBN1, FBN2, PLOD1, and PLOD2. Note that the representative aRMS IHC image for FBN1 shown is nearly negative (scoring is 1). Antibody specificity is with tumor specimen ID in parentheses listed on the figure. Models used for IHC include murine aRMS models (U21459, U24988, U48484, U20745, and U23674), a human aRMS model (pcb00380), murine eRMS models (U37125, U34278, U24085, and U24055), and a human eRMS model (pcb00232).

Given that RNA studies were conducted on whole tumor, which includes tumor cells and stromal cells, we sought to understand and further differentiate the source of these collagen and collagen-associated gene product expressions by immunohistochemistry on custom tissue microarrays. The expressions of COL18A1, PLOD1, PLOD2, FBN2, COL4A1, and COL4A2 were present in both murine aRMS and murine eRMS (**Table 1** and **Figure 5**). However, FBN1 was not expressed in murine aRMS and was weakly expressed in only one model of murine eRMS (**Table 1** and **Figure 5**). Models presented as representative images for IHC results include murine aRMS models (U21459, U24988, U48484, U20745, and U23674), a human aRMS model (pcb00380), murine eRMS models (U37125, U34278, U24085, and U24055), and a human eRMS model (pcb00232). The complete set of 48 murine RMS models and four human RMS models used for IHC analysis and associated scores are provided in **Supplementary Table 3**. Undifferentiated sarcomas, which include a variety of morphologic phenotypes from spindled to epithelioid and pleomorphic expressed all collagen-modifying enzyme to varying degrees (**Table 1**). No statistically significant trends were found in human RMS due to small sample size. Given the visual similarity between staining of extracellular matrix proteins and IHC background, a cohort of positive control images showing no background when used on skeletal muscle tissues are provided (**Supplementary Figure 1**).

**Table 1:** Immunohistochemical expression of collagens, fibrillins and collagen modifying enzymes in human and murine models.

	COL18A1	COL4A1	COL4A2	FBN1	FBN2	PLOD1	PLOD2
**Human Model**							
aRMS	100% (1/1)	0% (0/1)	100% (1/1)	0% (0/1)	0% (0/1)	0% (0/1)	100% (1/1)
eRMS	67% (2/3)	33% (1/3)	67% (2/3)	50% (1/2)	100% (3/3)	33% (1/3)	100% (3/3)
**Mouse Model**							
**Rhabdomyosarcomas**	69% (20/29)	79% (23/27)	14% (4/29)	4% (1/22)	66% (19/29)	48% (14/29)	72% (21/29)
aRMS	86% (12/14)	93% (13/14)	6.7% (1/15)	0% (0/11)	64% (9/14)	71% (10/14)	79% (11/14)
eRMS	67% (4/6)	83% (5/6)	67% (4/6)	17% (1/6)	67% (4/6)	17% (1/6)	67% (4/6)
Lineage Origin:							
Early Myoblast (Myf5)	70% (7/10)	89% (8/9)	10% (1/10)	0% (0/8)	60% (6/10)	50% (5/10)	80% (8/10)
Postnatal Stem Cell (Pax7)	41% (7/17)	63% (10/16)	13% (1/8)	9% (1/11)	63% (10/16)	18% (3/17)	35% (6/17)
Maturing Myoblast (Myf6)	75% (12/16)	88% (14/16)	18% (3/17)	9% (1/11)	71% (12/17)	59% (10/17)	69% (11/16)
**Undifferentiated Sarcomas**	50% (7/14)	64% (9/14)	14% (2/14)	10% (1/10)	64% (9/14)	24% (4/17)	29% (4/14)

aRMS, alveolar rhabdomyosarcoma; eRMS, embryonal rhabdomyosarcoma.

Comparison of RMS *versus* undifferentiated sarcomas in mice showed a significantly higher expression of PLOD2 in RMS (p = 0.044). When comparing aRMS to eRMS, a significantly higher expression of PLOD1 was seen in the aRMS subtype (p = 0.05) (**Table 1**). In general, murine sarcomas with *Rb1* nullizygosity were associated with the undifferentiated morphology, except for one case identified as aRMS which also showed lower expression of COL18A1, COL4A1, and PLOD2 compared to *Rb1* wildtype sarcomas (p = 0.0035, 0.04, and 0.08, respectively).

When comparing samples based on cell-of-origin (early myoblast, postnatal stem cell and maturing myoblast), a significant increase in PLOD1 was seen in cases of early myoblast and maturing myoblast origin RMS compared to the postnatal stem cell origin (p = 0.04). No other significant differences in cell or lineage-of-origin were seen across the other IHC markers.

**Figure 6:**
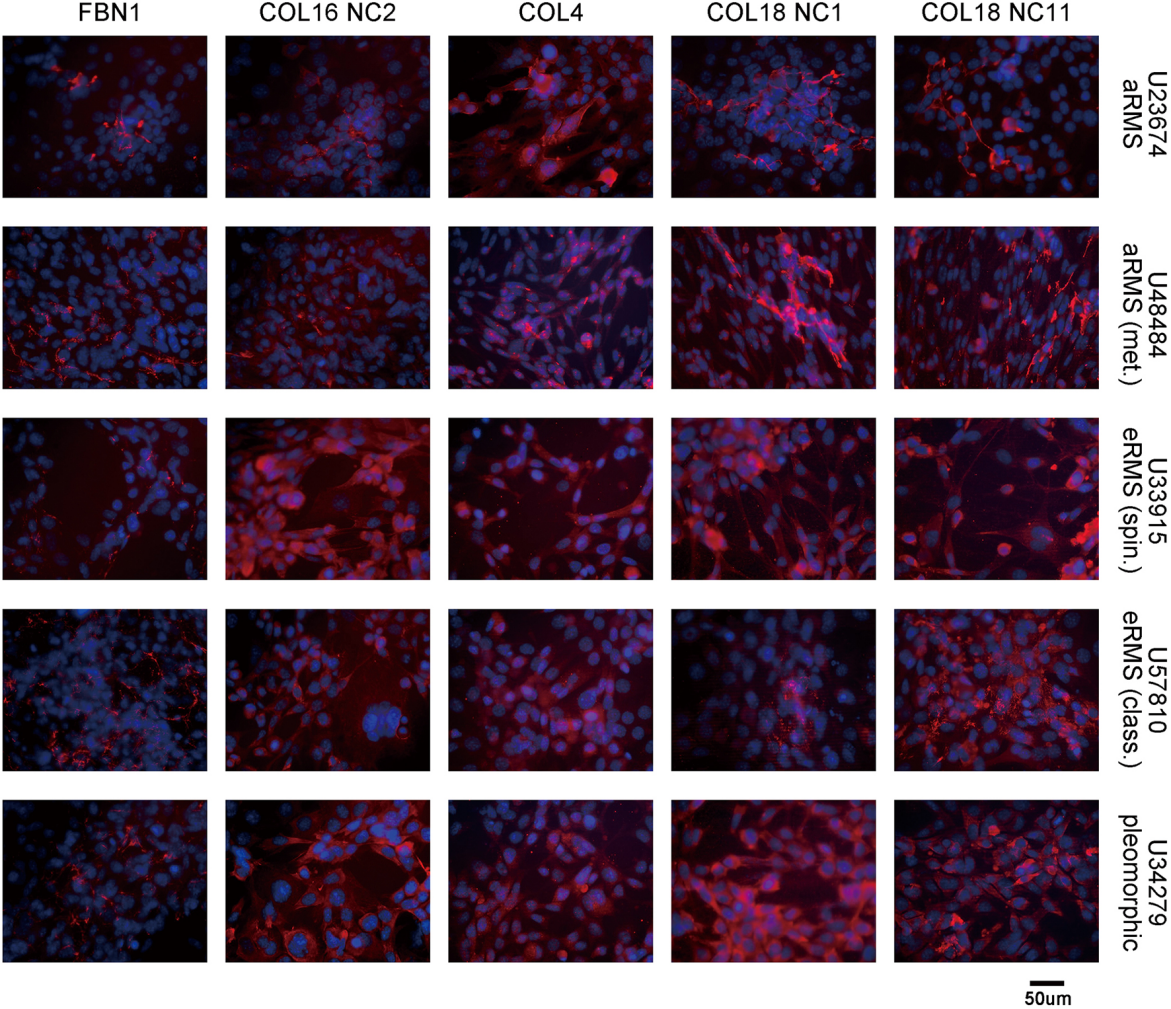
Immunocytochemical detection of ECM-related proteins in murine sarcoma models. Expression of ECM proteins in primary cell cultures derived from murine RMS. Immunocytochemistry using five independent antibodies against fibrillin-1, collagen XVI, collagen IV and two antibodies recognizing NC1 and NC11 domains of collagen XVIII. For each antibody, staining was intracellular and/or extracellular. Murine cell cultures used for ICC included U23674 (murine aRMS), U48484 (murine metastatic aRMS), U33915 (murine spindle cell eRMS), U57810 (murine eRMS), and U34279 (murine pleomorphic sarcoma).

**Figure 7:**
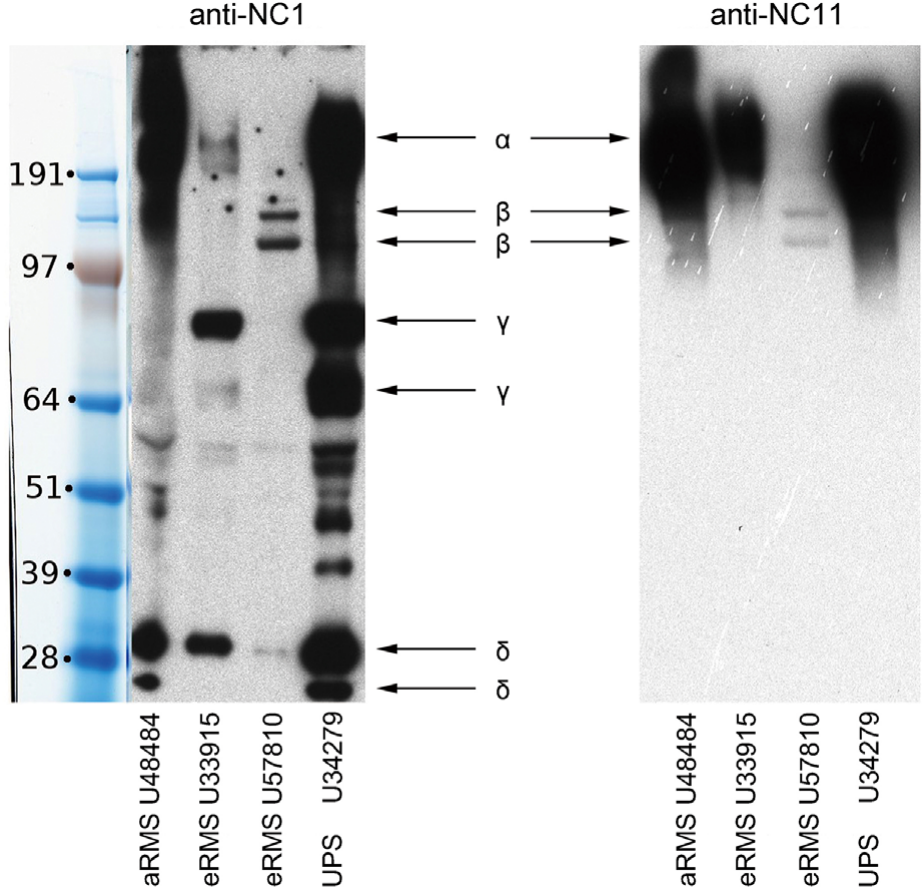
Biochemical analysis of secreted collagen XVIII in murine sarcoma models. Secretion of soluble type XVIII collagen molecules in cell cultures derived from murine RMS. Western-blot analysis of conditioned media from four cultures with two antibodies against type XVIII collagen. Antibody anti-NC1 recognizes the C-terminal non-collagenous domain (left panel), whereas antibody anti-NC11 recognizes the N-terminal non-collagenous domain (right panel). The bands depicted by arrow *α* are intact collagen XVIII. The bands depicted by arrow *β* are presumed collagen XVIII without heparan-sulphate. The bands depicted by arrow *γ* are presumed fragments of collagen XVIII without NC11 domain. The bands depicted by arrow *δ* are presumed endostatin/endostatin-containing fragments.

To visualize the intracellular *versus* extracellular localization of the ECM related protein in RMS cell lines, we performed immunocytochemistry (ICC) across several cultured murine RMS cells which showed expression of FBN1, the NC2 domain of COL16, COL4A1 as well as the NC1 and NC11 domains of COL18A1 (**Figure 6**). Murine cell cultures used for ICC included U23674 (murine aRMS), U48484 (murine metastatic aRMS), U33915 (murine spindle cell eRMS), U57810 (murine eRMS), and U34279 (murine pleomorphic sarcoma). Intracellular and secreted (extracellular) COL18A1 was readily detectable by ICC (**Figure 6**). Secreted COL18A1 in the conditioned media was also detected by immunoblotting (**Figure 7**).

### Clinical Significance of the Expression of COL18A1

**Figure 8:**
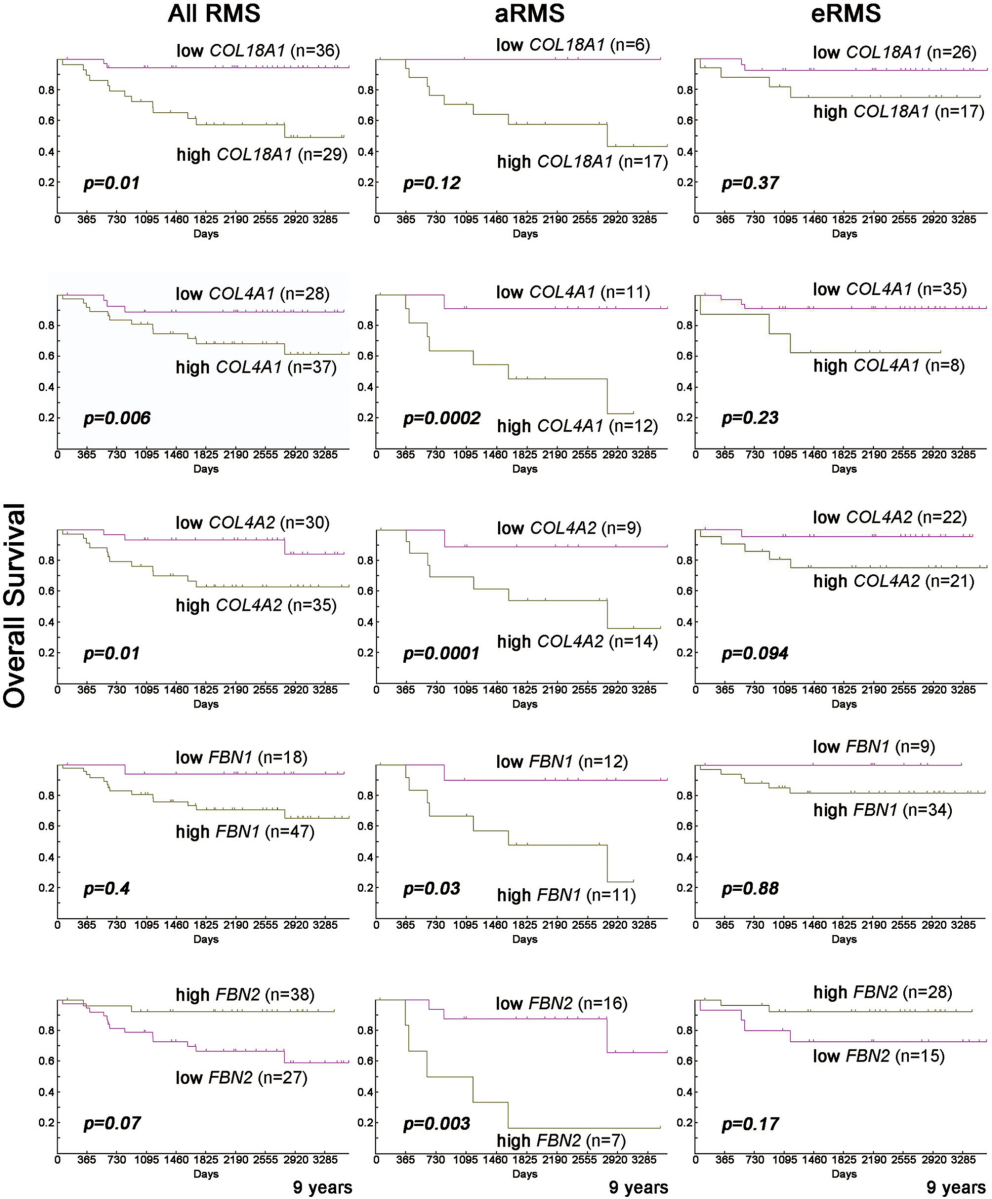
Overexpression of *COL18A1*, *COL4A1* and *COL4A2* at the RNA level is associated with worsened outcome. Decreased overall survival at 9 years for human RMS patients with elevated expression of *COL18A1*, *COL4A1* and *COL4A2*, even when adjusted for other known clinical covariates [as per analysis in (27)].

Biopsy samples were studied for gene expression analysis as previously described (27). In the biopsy samples provided by the Intergroup Rhabdomyosarcoma Study-IV (IRS-IV) (27), higher expression of COL18A1, COL4A1, and COL4A2 was correlated with worse outcomes in human RMS patients (**Figure 8**), with the alveolar subtype showing worsened survival compared to other RMS subtypes (**Figure 8**). Additionally, increased expression of FBN1 and FBN2 was correlated with worsened survival in aRMS (**Figure 8**); however, statistical significance is not seen for FBN1 and FBN2 when examining all RMS (**Figure 8**). IRS-IV samples used for analysis consisted of every available aRMS and eRMS sample in the IRS-IV database for which expression data was available. Samples available differed between groups.

## Discussion

The structural integrity of collagen fibers is reliant on a balance of post-translational modification and extracellular matrix enzyme composition, which can be significantly altered in tumors. To date, studies of the RMS TME are limited. However, mutations in *COL2A1* have recently been associated with chondrosarcoma risk (28).

As individual prognostic markers, increased COL18A1, COL4A1, and COL4A2 expressions are all associated with decreased long-term survival in all pediatric RMS subtypes, with the alveolar subtype showing worsened survival *versus* other subtypes. aRMS is most often fatal when metastatic (29), yet recent studies of related soft tissue sarcomas suggest that collagen subtype and modification can determine the ease with which these sarcomas metastasize (14); furthermore, altering collagen modifications is therapeutically amenable with FDA-approved agents that suppress metastasis in mouse soft tissue sarcoma models. From the perspective of therapeutic opportunities, another study has introduced a 3D murine model recapitulating the *in vivo* structure of aRMS and the ECM, which has promising possibilities for tumor behavior and therapeutic exploration (30).

In this study, we have shown that RMS expresses specific collagen proteins (COL18A1, COL4A1) at significantly increased levels in pediatric RMS relative to normal muscle; this expression in turn represents a worse prognosis (*COL18A1*, *COL4A1*, *COL4A2*). Strong IHC expression of COL18A1 was shown to have a worse outcome in pulmonary carcinomas (31) similar to *COL18A1* RNA expression in RMS. Moderate to strong COL18A1 IHC staining is also seen in greater than two-thirds of murine and human RMS samples in our study. Due to small sample size in human RMS, statistical analysis is limited to data generated from the murine RMS cohort. The dysregulated expression of COL18A1 and the worse prognosis associated with increased expression raises the possibility that COL18A1 mediates RMS metastasis. Furthermore, modification or cleavage of COL18A1 (*e.g.* endostatin) perhaps alters tumor biology *e.g.*, dysregulating tumoral angiogenesis or increasing metastatic potential (32). In our exploration of tumoral production of collagen, we also sought to explore the possibility of RMS utilizing enzyme production to alter the collagen matrix of surrounding tissues. This phenomenon in theory is an efficient way for a tumor originating in a stromal environment to quickly spread locally and gain access to blood supply/nutrition. Both *PLOD1* and *PLOD2* were shown to have increased overall RNA expression in human RMS relative to normal muscle. PLOD1 is an enzyme responsible for catalyzing the hydroxylation of lysyl residues in collagen-like peptides and is known to be deficient in patients with kyphoscoliotic form of Ehlers–Danlos syndrome (33). Although PLOD1 protein expression was present in less than 50% of RMS overall, a significantly larger number of murine aRMS samples showed PLOD1 expression compared to eRMS; however, this finding did not correlate with RNA expression patterns as *PLOD1* expression in both murine aRMS and eRMS is statistically indistinguishable. A significant increase in *PLOD1* RNA expression was seen in both human aRMS and eRMS relative to normal muscle; unexpectedly, PLOD1 protein expression is significantly higher in eRMS than aRMS.

Previous studies have shown correlation of PLOD2 expression with metastatic potential in soft tissue sarcoma (14) akin to our discoveries in RMS. The fact that a larger number of aRMS show increased PLOD1 expression compared to eRMS may be related to the possibility that the more aggressive alveolar subtype utilizes hypoxia-dependent mechanisms to break down collagen. However, *PLOD1/2* RNA expression did not correlate with worse outcome when looking at all RMS samples as a cohort and adjusting for stage.

In an attempt to isolate a specific tumoral mechanism based on the myogenic cell-of-origin, we analyzed whole transcriptome sequencing data segmented across murine aRMS and eRMS, and protein expression by IHC in RMS segmented by disease indication and by cell-of-origin (early myoblast, postnatal stem cell and maturing myoblast). Protein expression analysis by IHC yielded one important finding: significantly fewer cases of RMS with postnatal stem cell as cell-of-origin showed PLOD1 positivity compared to other cells-of-origin. Given that cell-of-origin influences pharmacological response (5), absence of PLOD1 positivity could potentially have diagnostic or even therapeutic value. Note that in the context of murine RMS models which have undergone whole transcriptome sequencing, the cell-of-origin status cleanly separated by histological diagnosis of aRMS or eRMS. Thus, we did not perform cell-of-origin based statistical comparison of extracellular matrix protein gene expression.

A more aggressive, undifferentiated morphology was associated with the *Rb1* nullizygous mouse sarcomas and was not seen in eRMS subtypes. The *Rb1* nullizygous sarcomas had a significantly lower number of tumors expressing COL18A1, COL4A1, and PLOD2. As described above, expression of COL18A1 and PLOD2 is associated with increased tumor infiltration, metastasis, and worse overall survival and thus offer potential therapeutic targets. This preliminary data suggests these targets are present in a higher number of aRMS/eRMS cases than in undifferentiated sarcomas and may represent a treatment opportunity.

Overall, our findings imply that RMS produces an imbalance in expression of a variety of collagens and collagen-modifying enzymes implicated in tumor growth and metastasis. COL18A1 expression is significantly increased in all RMS compared to normal muscle and is associated with worse overall survival. Our identification of overexpression of additional collagen markers at the RNA and protein level, specifically PLOD1 and PLOD2, may be considered for evaluation with potential FDA-approved or investigational therapies targeting these enzymes in future studies.

## Data Availability Statement

All sequencing data used to generate results in this manuscript have been previously deposited to online sequencing databases. The following datasets under the given accession IDs were used: GSE138269, GSM984615, GSM758578, GSE142775 (Gene Expression Omnibus database), EGAS00001004359, EGAS00001003981 (European Genome-phenome Archive), PRJNA613152 (Short Read Archive), Champions TumorGraft database, Mouse Tumor Biology Database (http://tumor.informatics.jax.org/mtbwi/pdxSearch.do), the OncoGenomics Database (https://pob.abcc.ncifcrf.gov/cgi-bin/JK), phs001121.v1.p1 (Database of Phenotypes and Genotypes). GTEx normal tissue gene expression data is available online through the GTEx portal (https://gtexportal.org/home/). Additional murine normal tissue expression data is available through the EBI Gene Expression Atlas (https://www.ebi.ac.uk/gxa/home).

## Author Contributions

JB, AM, and CK designed the study. XL, AB, TeS, SB, MC, EP, and SG performed experiments.; JS, ML, JB, and CH performed computational analysis. JS, MC, XL, NB, JB, CH and NEB analyzed and interpreted data. CK provided samples. TaS provided materials and validation of materials. JS provided bioinformatics data. XL, NB, NEB, JB, and CK wrote the manuscript. CK, HB, GS, and AM directed studies. All authors contributed to the article and approved the submitted version.

## Funding

This study was funded by Braver, Stronger Smarter Foundation, Brighter Days Childhood Cancer Organization, and Rub Out Rhabdomyosarcoma.

## Conflict of Interest

Authors ML and GS are employed by the company Omics Data Automation.

The remaining authors declare that the research was conducted in the absence of any commercial or financial relationships that could be construed as a potential conflict of interest.

## Supplementary Material

Supplementary Figure 1 Positive control skeletal muscle tissues stained for multiple ECM proteins.

Supplementary Table 1 Tissue microarray demographics and information.

Supplementary Table 2 Statistical tests for RNA sequencing expression comparisons between ECM-associated transcripts based on histopathological disease diagnosis.

Supplementary Table 3 Immunohistochemistry scores for each model across all tested analytes.
