# Supplementary figures and images for: Defining the Extracellular Matrix of Rhabdomyosarcoma
Source: Front Oncol. 2021 Feb 23;11:601957. doi: 10.3389/fonc.2021.601957 (PMC7942227; doi:10.3389/fonc.2021.601957)

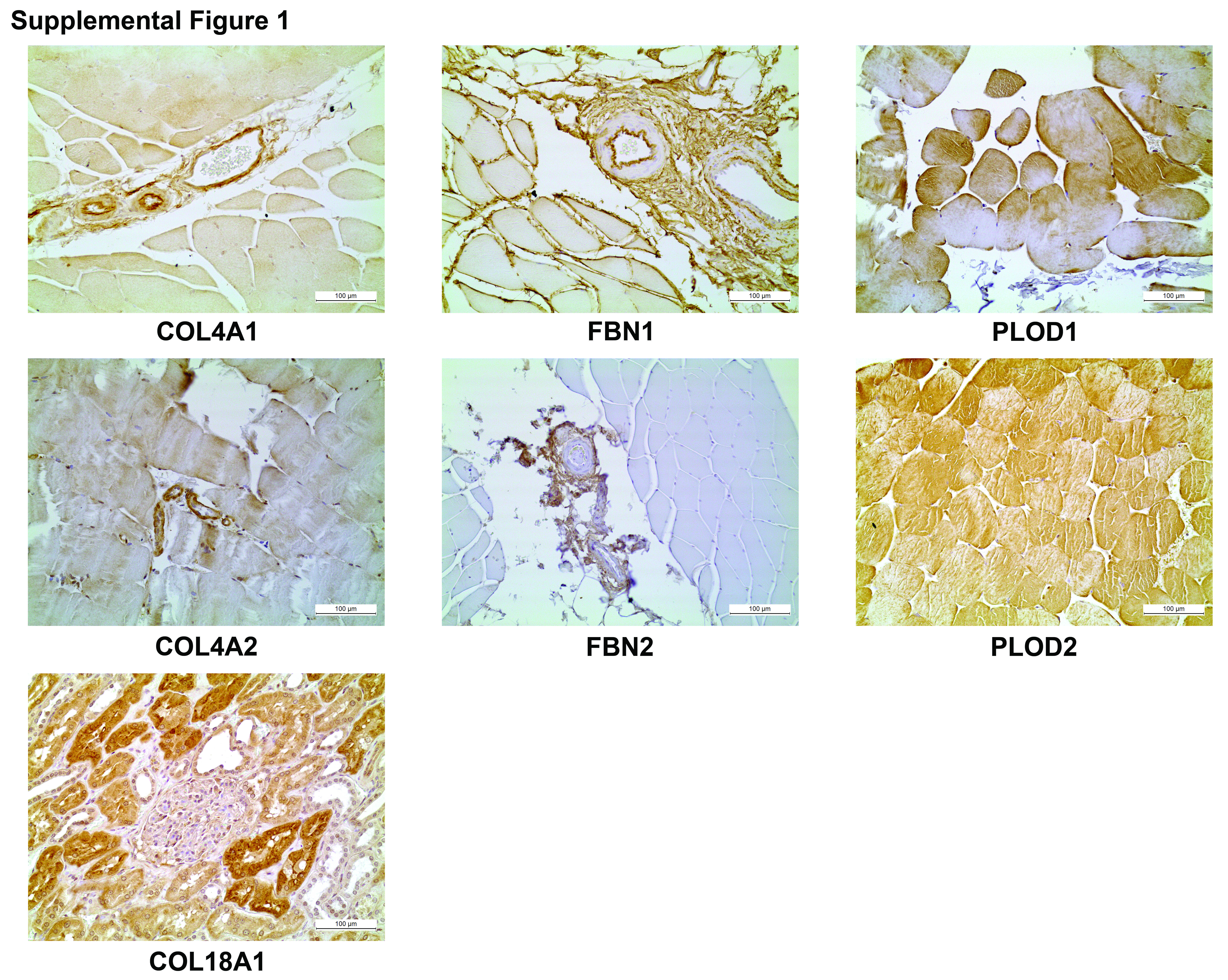

Supplement: Supplementary Figure 1 — Positive control skeletal muscle tissues stained for multiple ECM proteins. [file Image_1.jpeg]
